# Refractive Changes Induced by Strabismus Corrective Surgery in Adults

**DOI:** 10.1155/2017/2680204

**Published:** 2017-01-16

**Authors:** Daphna Mezad-Koursh, Ari Leshno, Tomer Ziv-Baran, Chaim Stolovitch

**Affiliations:** ^1^Department of Ophthalmology, Tel Aviv Sourasky Medical Center, Sackler Faculty of Medicine, Tel Aviv University, Tel Aviv, Israel; ^2^Goldschleger Eye Institute, Sheba Medical Center, Sackler Faculty of Medicine, Tel Aviv University, Tel Aviv, Israel; ^3^School of Public Health, Sackler Faculty of Medicine, Tel Aviv University, Tel Aviv, Israel; ^4^Assuta Medical Center, Tel Aviv, Israel

## Abstract

*Purpose*. To investigate refractive changes after strabismus correction procedures among adults.* Methods*. Retrospective chart review of adult patients who had horizontal recti muscles surgery with preoperative and postoperative cycloplegic refraction measurements. The preoperative refraction was mathematically subtracted from the postoperative refraction, and the induced refractive changes were statistically analyzed. Vector analysis was used to examine the magnitude of the toric change. The proportion of clinically significant refractive change was evaluated as well.* Results*. Thirty-one eyes from 22 subjects met the criteria and were included in the final analysis. A significant postoperative refractive change of the spherical equivalent towards myopia and a change of the astigmatism in the with-the-rule direction were observed. In a subset of 9 cases a third cycloplegic refraction measurement demonstrated stable refraction compared to the 1-month postoperative measurement. In 10 cases of single eye surgery, significant refractive changes were observed only in the operated side when compared to the sound eye. The induced surgical refractive change was of clinical significance (≥0.5 D) in 11 eyes of 9 patients (40.9% of patients).* Conclusions*. Refractive changes are a significant side effect of horizontal strabismus corrective surgery among adults. Therefore, patients should be informed about it prior to surgery and should be rerefracted in the postoperative period.

## 1. Introduction

The influences of strabismus surgeries on the refractive error have been investigated for many years; however, the methodology of the reports differs significantly with regard to the study population, surgical procedure, measuring methods, and statistical analysis.

Marshal, who was the first to describe in 1936 the relations between strabismus surgery and refractive change, reported a change in astigmatism in 60% of the patients [1]; later reports found a weaker association between strabismus surgeries and induced refractive error, with a leading finding of a transient astigmatic change after horizontal recti muscle surgery towards with-the-rule direction [2–7]. Though the majority of publications found no statistically significant change in spherical equivalent (SE) [2–5], there are reports of a transient myopic shift [5, 8, 9]. Hong and Kang [7] reported a significant change in SE towards myopia in a pediatric population operated for exotropia; however, since refractive measurements were performed preoperatively after cycloplegia and postoperatively without cycloplegia by using autorefractometer, these results might be the result of myopic shift due to strong accommodation in children.

There is no consensus among previous publications as to the clinical significance of the refractive changes [3, 5–7, 10–12]. Although the majority of strabismus surgeries are performed in the pediatric population, such study groups are far less than optimal for evaluation of refractive changes due to additional component of physiologic refractive change during childhood. We therefore believe that refractive changes after strabismus surgery in adults can reflect better the effect of the surgery itself on refraction without the confounder effect that exists in children.

The evaluation method of refractive changes induced by strabismus surgery is not uniform in previous studies. The first studies in the 1980s [13, 14] focused on astigmatic changes and described differences between the preoperative and postoperative refractive parameters. Later studies used statistical analysis tests for statistical significance [2, 6, 8]. In an attempt to quantify the extent of refractive changes, several approaches were used: Preslan et al. [3] calculated refractive changes induced by the surgery by simple subtraction of the cylinder powers, using one sign convention independent of axis, before and after the procedure. Since this form of analysis gives no information on surgical events in the cornea, the “law of sines and cosines” [15], described by Jaffe and Clayman [16], or power vector analysis [7] was used to calculate surgically induced astigmatism (SIA). Recent studies [2, 6, 9] used vector analysis methods described by Holladay et al. [17] or the Alpins methodology [18].

Most of the previous studies included children and adults [2, 3, 5, 9, 10, 14, 15]. While there are several publications dealing with children only [6–8] we did not find any large series of refractive change after strabismus surgery in adults only.

The purpose of this study is to evaluate the change in refractive error after strabismus surgery in adults by using the mathematical methods for analyzing and reporting aggregate surgically induced refractive changes (SIRC) as described by Holladay et al. [17]. SIRC calculation is a trigonometric method for calculating the exact spherocylindrical difference between the preoperative and postoperative refractions. In addition to the statistical significance of the refractive change, we were interested in evaluating the proportion of clinically significant (≥0.5 Diopter of change) refractive changes as well. The rationale of reviewing adults' cases only is their relative stable refractive state and a lower probability for physiologic refractive change compared to children.

## 2. Methods

### 2.1. Patient Selection and Data Collection

This retrospective study was approved by the institutional review board of Assuta Medical Center, Tel Aviv, Israel, and was fully compliant with the principles of the Declaration of Helsinki.

We retrospectively reviewed all charts of patients treated at the senior author of this article's clinic (Chaim Stolovitch) who have undergone horizontal strabismus corrective surgery at the age of 18 or older. All procedures where performed by one of the authors (Chaim Stolovitch). Cases were included if the chart contained refraction examination measurements both preoperatively (up to 3 months prior to surgery) and one month postoperatively. Exclusion criteria included a history of ocular surgery in the 3 years prior to the current operation, congenital or progressive corneal disease, familial or acquired posterior segment disease, glaucoma, a history of ocular trauma, or neurological or systemic disease, operation on vertical recti or oblique muscles, and any surgical complication.

Cycloplegic refraction was performed 40 minutes after instillation of 1% cyclopentolate twice, 2 minutes apart [19]. All refractions were performed by the senior author of this article (CS) by using a hand-held retinoscopy in a darkened room and subjective refinement of the prescription by using cross cylinder as needed. All surgeries were performed between May 2003 and August 2013.

For each patient the following data was reviewed: age of patient at time of surgery, sex, motor alignment before and after the surgery at near and distance fixation, type of surgery performed, refraction before and after the surgery, and best corrected visual acuity before and after the surgery.

### 2.2. Statistical Analysis

In order to evaluate the surgical induced refractive changes (SIRC), the difference between each postoperative refraction and the respective preoperative refraction was calculated using double-angle mathematical methods for subtraction of refractions, which were first described by Naylor [20] and further developed by Holladay et al. and Retzlaff et al. [21–23]. Statistical analysis of the aggregate data was performed according to the methods further described by Holladay et al. [17]. We compared postoperative refractive measurements with the corresponding preoperative value by applying paired* t*-test and Wilcoxon signed-rank test for normally and nonnormally distributed variables, respectively. In addition, cases were stratified and compared according to astigmatism at presentation and number of muscles involved in the operation, using Man-Whitney and Student's* t*-test as necessary. Fisher's exact test was used for categorical variables. Influence of the extent of muscle change on SIRC was evaluated using Spearman's correlation. In cases of two operated muscles the muscle in which the change was greater was used.

Lastly we evaluated the clinical significance of the changes. A change in refractive power of 0.5 D or more was considered clinically significant. One-sample binomial test was used to evaluate whether the proportion of clinically significant changes was greater than an acceptable 10%. All statistical analyses were performed using IBM SPSS statistics 22.0 program (IBM SPSS Statistics for Windows, Version 22.0. Armonk, NY: IBM Corp.). A *P* value less than 0.05 was considered statistically significant.

### 2.3. Example of the Double-Angle Mathematical Methods for Subtraction of Refraction

In order to determine the SIRC we used a modification of the rectangular coordinate method described by Holladay et al. [21] to determine whether the SIRC was applied. In short, SIRC can be determined by adding the negative of the preoperative refraction (PreRx) to the postoperative refraction (PostRx) by following these 10 steps:

Formula will be followed by numerical example:

(1) SIRC=PostopRx−PreopRxSC1=Sph1+Cyl1×Axis1SC2=Sph2+Cyl2×Axis2−PreopRx=−1.75−1.75×65PostopRx=+0.75+3.00×90.

**Step 1 .**

Transpose one of the spherocylinders so that the cylinders have the same sign:

(2) $$\begin{array}{c} -PreopRx=-3.50+1.75\times 155. \end{array}$$

**Step 2 .**

Spherocylinder 1 (SC1) is chosen so that Axis_1_ < Axis_2_

(3) SC1=PostopRx;SC2=−PreopRx.

**Step 3 .**

Find the angle ∝:

(4) ∝=Axis2−Axis1∝=155−90=65°.

**Step 4 .**

Find the angle 2*β*:

(5) tan 2βCyl2sin 2αCyl1+Cyl2cos 2αtan⁡2β1.75∗sin⁡2∗653.00+1.75∗cos⁡2∗65=0.72⟶2β=35.56°.

**Step 5 .**

Find angle *θ*:

(6) θ=2β+1802θ=35.56+1802=107.8°.

**Step 6 .**

Determine the sphere contributed (SC):

(7) SCCyl1sin2θ+Cyl2sin2α−θSC3.00∗sin2⁡107.8+1.75∗sin2⁡65−107.8=3.53.

**Step 7 .**

Determine the total spherical result (Sph_3_):

(8) Sph3=Sph1+Sph2+SCSph3=−3.50+0.75+3.53=0.78.

**Step 8 .**

Determine the total cylindrical result (Cyl_3_):

(9) Cyl3=Cyl1+Cyl2−2SCCyl3=3.00+1.75−2∗3.53=−2.31.

**Step 9 .**

Determine the resultant axis (Axis_3_) in standard notation (if A3 > 180 subtract 180 for standard axis notation; if A3 is negative, add 180):

(10) Axis3=Axis1+θAxis3=90+107.8=197.8⟶Axis3=17.8°.

**Step 10 .**

If needed, formula can be transposed from (−) cylinder to (+) cylinder form.

(11) $$\begin{array}{c} SIRC=-1.53+2.31\times 107.8^{{}^{\circ}}. \end{array}$$

Since cylinder power and axis are not independent parameters, in order to determine the mean induced astigmatism the polar values of astigmatism were converted to Cartesian values using the two following equations:

(12) x=Cylinder∗cos⁡2 Axisy=Cylinder∗sin⁡2 Axis.

The mean cylinder power and angle were calculated using the formulas:

(13) Cylindermean=xmean2+ymean2Axis=0.5∗arctan ymeanxmean.

The SD of the cylinder (*s*_cyl_) was calculated as the square root of the SD of the set of *x*(*s*_*x*_) times the SD of the set of *y*(*s*_*y*_):

(14) $$\begin{array}{c} S_{cyl}=\sqrt{S_{x}*S_{y}}. \end{array}$$

## 3. Results

A total of 31 eyes from 22 individuals (9 males and 15 females) met the above criteria and were included in the final analysis. The median age of the patients was 31 years (range 18–48 years).  Table 1 depicts the baseline characteristics of the study group. All refractive measurements were done up to three months prior to surgery with the exception of 6 subjects (subjects 13, 14, 17, 19, 20, and 21) who were examined up to 10 months before the surgery and had no change in visual acuity measurements until the surgery. There were no surgical complications in any of the cases.

A summary of preoperative and 1-month postoperative SE is depicted in Table 2. The SE was significantly different between the two measurements (*P* < 0.0001). Statistical significance was maintained after stratification according to preoperative astigmatism or number of muscles involved in the operation.

**Figure 1:**
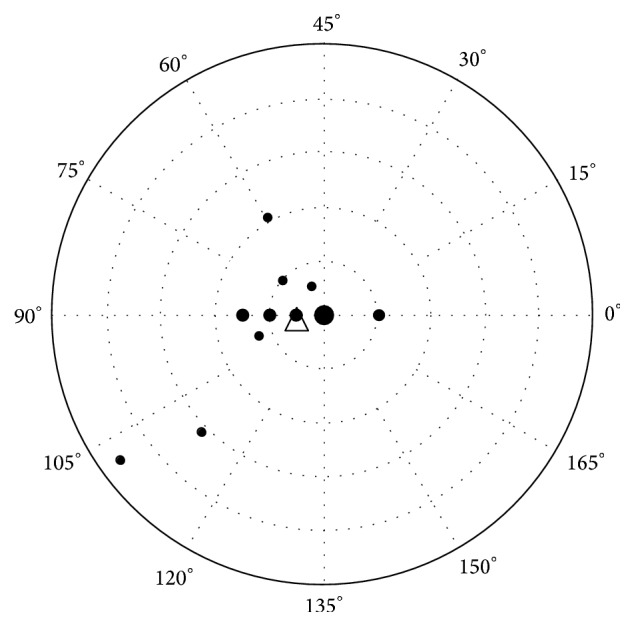
A double-angle, plus cylinder power plot for surgically induced astigmatism. ^*∗*^Rings equal 0.5 D steps (inner ring = 0.5 D; outer ring = 2.5 D). Size of points represents number of cases with same cylindric change. △ = centroid 0.25 ± 0.42 D × 94°.

Since the outcome of astigmatic correction depends on the axis as well as the magnitude of the toric change, vector analysis was used to examine these changes. After calculating the SIRC for each eye, the method described by Holladay and colleagues [17] for evaluating and reporting astigmatism for aggregate data was applied.  Table 3 summarizes the SIRC according to preoperative astigmatism and number of muscles involved in surgery. Overall, the magnitude of surgically induced cylinder was found to be significantly different from zero (0.25 ± 0.42, *P* < 0.005) as well as the induced SE (−0.30 ± 0.35, *P* < 0.001) (Table 3). Statistical significance was maintained after stratification according to preoperative astigmatism or number of muscles involved except for the induced cylinder in the one muscle subgroup. In addition, preoperative astigmatism was significantly associated with postoperative change in cylinder (OR 5.85 95% CI 1.22–27.99, *P* = 0.033).  Figure 1 illustrates surgically induced cylinder power using a double-angle plot.

In a subset of 9 cases a third cycloplegic refraction measurement was available from a subsequent follow-up visit (mean 814.6 ± 590 days after the surgery), which did not differ significantly from the 1-month postoperative measurement.

In order to validate the data we checked for refractive changes in contralateral eye, in patients operated unilaterally. Out of 13 subjects who had surgery in one eye only, 11 had both preoperative and postoperative refractive measurements for the sound eye as well. In one case (subject 11) the contralateral eye was operated a few weeks prior to the first refractive measurement and was therefore excluded. In the remaining 10 cases, the mean surgically induced SE was significant only in the operated eye (−0.42 ± 0.15 D, *P* = 0.007) while changes in the other eye remained within the margin of error (−0.23 ± 0.44, *P* = 0.336).

**Figure 2:**
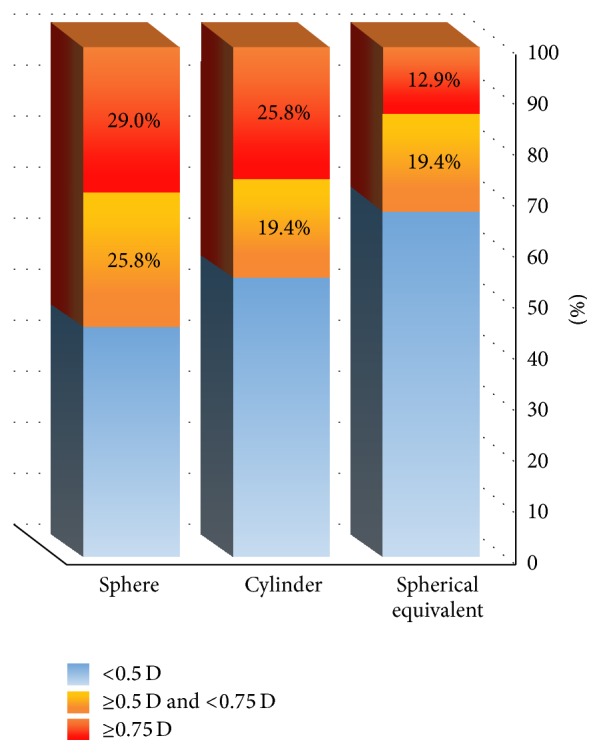
Proportion of cases in which the surgically induced change in refraction was clinically significant.

We observed a substantial proportion of cases with clinically significant refractive changes (≥0.5 Diopter of change) after surgery.  Figure 2 depicts the proportion of cases in which the refractive change was of clinical significance in each parameter. Using one-sample binomial test we found all proportions to be statistically significant (*P* < 0.0001) for changes equal or greater than 0.5 D as well as for changes equal or greater than 0.75 D. Statistical significance was maintained in all subgroups after stratification according to preoperative astigmatism or number of muscles involved. A new prescription for glasses was needed after the surgery to regain preoperative BCVA in eleven cases and the need for new prescription was significantly associated with surgically induced SE change (*P* = 0.037). The extent of maximal muscle change correlated with surgically induced SE change (Spearman's rho −0.454, *P* = 0.010); however, this correlation was not maintained in the subgroup analysis.

## 4. Discussion

Although the majority of strabismus surgeries are performed in the pediatric population and the clinical importance of a lasting postoperative refractive change is relevant mainly in this population, we chose to focus on the adult population in this study. The refractive power of the growing eye changes dramatically during the first years of life [24]; axial length increases up to the age of 13, cornea reaches the adult power by the age of 12, and the power of the pediatric lens decreases dramatically in the first decade of life. We therefore assumed that refractive changes after strabismus surgery in adults reflect better the effect of the surgery itself on refraction without a possible additional component of physiologic refractive change as in childhood.

Similar to previous studies [2–7] we found a significant change in astigmatism in the with-the-rule direction in the cylinder magnitude and axis towards with-the-rule direction. As one can see in Tables 2 and 3; the refractive changes proved to be statistically significant. Interestingly, preoperative astigmatism was strongly associated with clinically significant surgically induced cylinder when compared to a subgroup without preoperative astigmatism.

As our main research question regards the clinical significance of the refractive change, we set a change of at least 0.5 D as a clinically significant change.

As shown in Figure 1 the surgically induced astigmatism for most patients is within a 1 D of astigmatism; however, 3 patients had an astigmatic change larger than 1 D and in one patient the astigmatic change was larger than 2 D.

We also presented separately in Figure 2 the proportion of cases in which refractive change was clinically significant. In 33.2% of our patients the SE change was ≥0.5 D, a change in the cylinder power alone and in sphere power alone of ≥0.5 D was observed in 45.2% and 54.8% of the patients, respectively, and 35.5% of the patients needed a new prescription for glasses. In a subset of 10 patients who had a unilateral surgery we were able to compare between the operated eye and the sound eye. In this “case-control” subgroup the SE changed significantly not only statistically, but also clinically in the operated eye and not in the sound eye

A major disagreement among previous publications regards the duration and therefore clinical significance of the refractive changes. While some investigators concluded that the refractive error change was transient [5, 10], others reported a long lasting clinically significant refractive change [3, 6, 7, 11, 12]. In some studies the absolute refractive change was not found significant but there was a clinically significant change in a subset of patients; Nardi et al. [10] concluded that the change in refractive error after horizontal muscle surgery is transient and insignificant, although they found residual astigmatism of >1 D and of >0.5 D at 30 days post-op among 6% and 12% of their patients, respectively. Rajavi et al. [5] also concluded the refractive change to be nonsignificant although 16% of their patients had astigmatic power change equal or more than 1 D 3 months after the surgery. Schworm et al. [15] reported a lasting induced astigmatism of more than 3 D in 4% of their patients 3 months after the surgery. Unfortunately, we do not have a longer than one-month follow-up for all our cases as many patients do not return for further examination after strabismus had resolved and there are adult patients who object to further cycloplegic examinations. In a subgroup of 9 patients (50%) having a third cycloplegic refraction performed at least 200 days postoperatively (mean of > 2 years after the surgery) we found the operative induced change to be stable.

According to the results of this study, performed on adults, in whom we consider the refraction to be stable, refractive error after strabismus surgery changes significantly. Although the mean change is not clinically significant, there was a clinical significant change in 50% of the patients. One can postulate that the same change occurs in the pediatric population, in which clinically significant change in refraction might interfere with binocular vision and amblyopia treatment or even cause amblyopia.

This is the first report regarding refractive change in adults. The results of this study should be interpreted within the context of its limitations. This is a retrospective review and it is subject to the selection and follow-up bias inherent to all retrospective reviews. Because this series of cases include mixed types of strabismus surgeries, we cannot suggest a common mechanism for the refractive change we found. Several mechanisms were offered to explain the change in refraction. Kushner [14] demonstrated that oblique muscle surgery produce a long-term cyclotortion which leads to a suitable change in the cylinder axis, we therefore excluded any case in which oblique or vertical recti muscles were operated on the same time. The main theory regarding the change in the spherical power and cylinder power and axis relates to corneal changes [1, 3, 11, 13, 25, 26].

Though this is a retrospective study, we were able to establish control group for part of the eyes, using the sound eye's refraction and to document a longer follow-up for another subgroup of patients. Unfortunately, those were smaller groups of patients.

In spite of its limitations, this study represents the largest series of refractive change secondary to strabismus surgery in adults. We agree with previous reports [3, 6, 7, 11] that refractive changes might be long lasting; however, a prospective research is needed in order to prove that.

## 5. Conclusion

Myopic shift and induced change of the astigmatism in the with-the-rule direction are significant side effects of strabismus corrective surgery among adults. The mean change is not clinically significant but there is a clinical significant change in 50% of the patients, which might be long lasting. Therefore, patients should be informed about this possible side effect prior to surgery and should be rerefracted in the postoperative period.

## Figures and Tables

**Table 1:** Baseline characteristics of study population.

Patient number	Age (yrs)	Sex	Eye	Procedure type	Preoperative refraction				Postoperative refraction			
					Sphere	Cylinder	Axis	Spherical equivalent	Sphere	Cylinder	Axis	Spherical equivalent
1	41	Female	RE	MR recession	−4.50	+0.75	75	−4.13	−4.50	+1.00	70	−4.00
			LE	MR recession	−3.50	+1.00	95	−3.00	−3.50	+0.50	100	−3.25
2	26	Female	RE	MR recession	−2.75	0.00	0	−2.75	−3.00	0.00	0	−3.00
			LE	MR recession	−2.75	0.00	0	−2.75	−3.25	0.00	0	−3.25
3	31	Female	RE	MR recession	+2.50	+2.50	60	+3.75	+2.50	+2.50	60	+3.75
			LE	MR recession	+1.00	+3.00	110	+2.50	+1.00	+3.00	100	+2.50
4	42	Female	RE	MR recession	−2.50	0.00	0	−2.50	−3.00	0.00	0	−3.00
			LE	MR recession	−2.50	0.00	0	−2.50	−2.50	0.00	0	−2.50
5	31	Male	LE	LR resection + MR recession	−3.75	+1.00	140	−3.25	−4.50	+1.25	125	−3.88
6	28	Female	LE	LR recession + MR resection	−0.25	+1.50	180	+0.50	−0.25	+1.50	180	+0.50
7	23	Male	RE	LR recession + MR resection	−8.25	+0.50	180	−8.00	−9.50	+1.25	120	−8.88
8	48	Female	RE	LR recession + MR resection	+0.50	+0.75	180	+0.88	0.50	0.00	0	+0.50
9	21	Male	RE	MR recession	−5.50	0.00	0	−5.50	−5.50	+0.50	70	−5.25
			LE	MR recession	−2.50	+0.50	90	−2.25	−2.75	+0.75	90	+2.38
10	29	Male	RE	LR recession + MR recession	−0.50	0.00	0	−0.50	−0.75	0.00	0	−0.75
11	47	Male	LE	LR recession + MR resection	−3.50	+3.50	30	−1.75	−3.50	+3.50	30	−1.75
12	20	Female	RE	LR recession + MR recession	−3.00	+0.50	90	−2.75	−3.00	0.00	0	−3.00
13	31	Male	RE	MR recession	−0.50	0.00	0	−0.50	−1.00	+0.75	90	−0.63
			LE	MR recession	+0.75	+0.50	180	+1.00	+0.50	+0.50	180	+0.75
14	24	Female	RE	MR resection	+1.25	0.00	0	+1.25	+1.00	0.00	0	+1.00
			LE	MR resection	+2.00	0.00	0	+2.00	+2.00	0.00	0	+2.00
15	35	Female	LE	LR recession + MR resection	−7.00	+3.00	90	−5.50	−7.75	+3.50	90	−6.00
16	37	Male	RE	MR recession + LR resection	+1.75	+1.75	65	+2.63	+0.75	+3.00	90	+2.25
17	37	Female	RE	LR resection + MR postfixation suture	+4.00	+0.75	90	+4.38	+2.75	+1.00	90	+3.25
			LE	LR resection	+4.75	0.00	0	+4.75	+3.00	+0.75	90	+3.38
18	20	Female	LE	LR recession + MR resection	+1.25	0.00	0	+1.25	+0.75	0.00	0	+0.75
19	18	Female	RE	LR recession	0.00	0.00	0	0.00	−0.50	0.00	0	−0.50
			LE	LR recession	0.00	0.00	0	0.00	−0.25	+0.50	180	0.00
20	47	Female	RE	LR recession + MR resection	+3.25	0.00	0	+3.25	2.75	+0.50	90	+3.00
21	20	Female	LE	MR recession + LR resection	−5.00	+0.75	90	−4.63	−5.50	+1.25	90	−4.88
22	18	Male	LE	MR recession + LR resection	−0.75	+0.50	90	−0.50	−1.00	−0.75	90	−0.63

**Table 2:** Comparison of preoperative and postoperative refractive power in terms of spherical equivalent (SE).

Grouping variable	Preoperative SE (mean ± SD)	Postoperative SE (mean ± SD)	*P* value^a^
Preoperative astigmatism			
Present (*N* = 17)	−1.18 ± 3.47	−1.48 ± 3.52	**0.002**
Absent (*N* = 14)	−0.32 ± 2.73	−0.63 ± 2.54	**0.011**
Muscles involved			
Single muscle (*N* = 18)	−0.62 ± 2.79	−0.83 ± 2.66	**0.021**
Two muscles (*N* = 13)	−1.04 ± 3.68	−1.45 ± 3.70	**0.001**
Total (*N* = 31)	−0.79 ± 3.14	−1.09 ± 3.10	**<0.0001**

^a^Paired samples *t*-test of preoperative compared to postoperative.

**Table 3:** SIRC according to preoperative astigmatism or number of muscles involved^a^.

Parameter (Diopter power), mean ± SD	Sphere	Cylinder^b^	Axis (degrees)^b^	SE	*P* value^c^	*P* value^d^
Preoperative astigmatism						
Present (*N* = 17)	−0.57 ± 0.52	0.35 ± 0.53	97	−0.29 ± 0.33	**0.015**	**0.002**
Absent (*N* = 14)	−0.41 ± 0.43	0.14 ± 0.17	85	−0.30 ± 0.39	**0.009**	**0.009**
Muscles involved						
Single muscle (*N* = 15)	−0.39 ± 0.43	0.11 ± 0.29	67	−0.26 ± 0.37	0.164	**0.010**
Two muscles (*N* = 16)	−0.60 ± 0.52	0.43 ± 0.48	100	−0.34 ± 0.34	**0.003**	**0.003**
Total (*N* = 31)	−0.50 ± 0.48	0.25 ± 0.42	94	−0.30 ± 0.35	**0.002**	**<0.001**

^a^Bold indicates value is statistically significant.

^b^Calculated using double-angle mathematical methods for subtraction of refraction.

^c^Surgically induced cylinder different from zero.

^d^Surgically induced SE different from zero.
